# Refining Reverse Shoulder Arthroplasty: From Implant Design to Patient-Specific Strategy

**DOI:** 10.3390/jcm15041361

**Published:** 2026-02-09

**Authors:** Stefan Bauer, William G. Blakeney, Allan W. Wang

**Affiliations:** 1 Centre de l’Épaule, du Coude et du Membre Supérieur, Service d’Orthopédie, Ensemble Hospitalier de la Côte, 1110 Morges, Switzerland; 2 Division of Surgery, Medical School, University of Western Australia, Perth, WA 6009, Australia; 3 Department of Orthopaedics and Trauma, Royal Perth Hospital, Perth, WA 6000, Australia; 4 Department of Orthopaedics and Trauma, St John of God Hospital, Murdoch, WA 6150, Australia

Reverse shoulder arthroplasty (RSA) has evolved from a reliable solution for cuff-deficient shoulders into a broadly utilized reconstructive replacement procedure [1,2,3,4] used in more than 80% of shoulder arthroplasty cases according to recent reports by the Australian registry [5]. With this growth has come increasing recognition of its inherent challenges, including limitations in postoperative external and internal rotation [6,7], the impact of scapular posture and motion [8,9,10,11,12], and the complex interplay between moment arms and muscle tensioning that influences both passive and active shoulder function [13,14,15,16,17].

These factors—alongside well-recognized complications such as instability [18,19,20,21,22,23,24,25,26], scapular notching [27,28,29,30,31,32,33,34,35], acromial and scapular spine stress fractures [36,37,38,39,40,41,42,43,44,45], and concerns about implant longevity [46,47,48] and functional performance [49,50]—underscore the need for patient-specific decision-making to reduce complications and optimize treatment [51,52,53,54]. Objective assessment of muscle quality [55,56,57] and imaging-based predictive modeling extend the patient-specific approach highlighted by Roche and colleagues in this Special Issue [58]. Collectively, the contributions to this Special Issue span infection revision, implant philosophy, fixation geometry, soft-tissue management, navigation technologies, biomechanics of instability, and outcome measurement.

A major practice-shaping contribution is provided by Collin, Lädermann, and colleagues [59], who evaluated outcomes following two-stage revision RSA for periprosthetic joint infection. By demonstrating functional results approaching those of primary RSA, this work reframes revision RSA as a restorative rather than salvage procedure. This constitutes an important shift for counseling and decision-making as revision burden rises [60,61,62].

Conceptual advancement is further driven by Frankle’s group [63], whose design journey in more anatomic RSA integrates biomechanical principles with clinical outcomes [64,65,66,67,68,69,70]. Their work challenges traditional assumptions regarding deltoid dependence and medialization, offering a coherent framework for understanding emerging implant philosophies rather than simply introducing another design iteration.

Moroder and colleagues [71] report five-year outcomes of a rectangular humeral stem with a 135° neck–shaft angle (NSA). These data underscore the importance of stem geometry and alignment, providing clinically meaningful guidance for implant selection.

Soft-tissue considerations are rigorously examined by Collin, Lädermann, and colleagues [72] in their comparison of onlay versus inlay RSA designs. By correlating implant configuration with subscapularis repair, healing, and functional outcomes, this work directly informs surgical technique in an area of longstanding debate [73,74,75,76,77,78,79].

Technological innovation is critically assessed by Kriechling, Wieser, and co-authors [80], who present a systematic review on augmented reality applications in RSA. Their analysis demonstrates improved accuracy and reproducibility of glenoid component placement while appropriately emphasizing the need for further clinical outcome validation before widespread adoption.

A mechanistically novel and clinically relevant phenomenon is described by Bauer et al. [81] in their investigation into instability patterns in short-stem RSA. They identify a distinct superior–lateral instability pattern associated with a 135° NSA, introduce the two-hand lever test (2HLT) as an intraoperative assessment, and highlight the importance of an effective NSA.Their findings suggest that lower angles, particularly 135°, may require increased liner constraint to maintain stability because of the direction of the deltoid force vector, especially in the presence of varus stem alignment and a more vertical joint line. Established concavity compression mechanics [82] gain a practical new dimension here: effective NSA as a modifiable parameter that can be optimized through multiple strategies [83].

Complementing these high-impact studies, works by Berhouet [84], Clauss [85], Gupta [86,87], Edwards [88], and colleagues add biomechanical insights, contemporary perspectives on infection and implant evolution, advanced glenoid and humeral reconstruction, and objective outcome assessment. In this context, advances with novel patient-reported outcome measures (PROMs) are promising in terms of capturing more relevant data on RSA performance beyond generalized pain and function [89].

In the past, researchers in this field aimed to achieve the best design. In the future, we may instead focus on identifying the best individual strategy, selecting from an expanding surgical armamentarium.
